# INTRA-ABDOMINAL SEMINOMA TESTIS IN ADULT: CASE REPORT

**DOI:** 10.1590/S0102-6720201500030021

**Published:** 2015

**Authors:** Jorge Roberto Marcante CARLOTTO, Ramiro COLLEONI-NETO, David Carlos SHIGUEOKA, Ricardo ARTIGIANI-NETO, Gaspar de Jesus LOPES-FILHO

**Affiliations:** 1 Surgical Gastroenterology Discipline, Department of Surgery; 2 Department of Diagnostic Imaging; 3 Department of Pathology of Paulista School of Medicine, Federal University of São Paulo, São Paulo, SP, Brazil

## INTRODUCTION

The cryptorchidism is the most common congenital malformation of the genitourinary tract^4^. The intra-abdominal testicle is subject to complications such as cancer, ischemia and infertility^1^. The most common malignant transformation of undescended testicle is the seminoma^2^^,^ 3 ^,^ 5. Here is presented a case of adult massive pelvic mass corresponding to seminoma developed in intra-abdominal testis. 

## CASE REPORT

**FIGURE 1:**
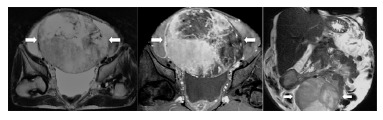
- MRI of the pelvis with heterogeneous mass with well vascularized with areas of degeneration/necrosis (arrows) and superior displacement of bowel loops

MC'S, man, 32, with a history of progressive and painless increased abdominal size four weeks ago, no other complaints. He had also several congenital malformations including: pectus excavatum, congenital dislocation of the hip and clubfoot (operated in childhood). Physical examination revealed ascites, bilateral pleural effusion and a hardened mass located in flank and the right iliac fossa of about 20 cm. Was detected the absence of the right testicle in scrotum. No abnormal laboratory tests were present. Computed tomography and magnetic resonance imaging of the abdomen and pelvis showed ascites and heterogeneous pelvic mass (Figure 1). The cytological study of ascites and pleural effusion showed no neoplastic cells.

**FIGURE 2:**
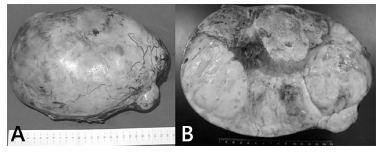
- Tumor mass with smooth outer surface, opaque, sometimes lobed, with brown bleeding areas in A. When cut, in B, whitish, firm and elastic tumor, permeated by areas of bleeding and yellowish and softened areas.

 Laparotomy was performed which identified a large solid mass in the right iliac fossa partially adhered to the right inguinal canal, and dry easily. The surgical specimen measured 25x19x12 cm and weighed 2350 g (Figure 2). Histopathological examination revealed seminoma of undescended testis with extensive area of coagulation necrosis and angiolymphatic neoplastic embolization.

The patient was discharged on the sixth day after surgery. The left testicle was evaluated and was normal. The staging was completed and once considered the patient in stage III (ascites), was subjected to four cycles of chemotherapy with bleomycin, etoposide and cisplatin, keeping normalization of markers and improvement of ascites. He is currently with eight years of evolution, and in that period maintained regular outpatient follow-up showing no measurable disease to blood tests and imaging.

## DISCUSSION

The cryptorchidism results from abnormalities in the formation and testicular descent during the embryonic period^4^. It is present in 6% of newborns at term and in 0.8% of infants under one year of age. Can be bilateral in up to 10% of cases, and sometimes is associated with other defects in genitourinary tract^5^.

The most feared complication of undescended testicle is cancer, ranging from 3.5-14.5% among patients with cryptorchidism^5^. The testis are intra-abdominal in 10% of cases and at risk 200 times greater in malignant transformation^3^. Malignant degeneration has the peak incidence in third and fourth decade of life^2^^,^ 3. They are usually asymptomatic and are identified incidentally by imaging tests. When symptomatic, diagnosis is difficult and the symptoms may mimic acute appendicitis, urinary calculus and mass effects, compressive symptoms of the gastrointestinal and genitourinary tracts^3^. Imaging tests, US, CT and MRI show pelvic or retroperitoneal mass, well defined, homogeneous, without obvious evidence of necrosis or calcification^2^^,^ 3 ^,^ 5. These findings have as main differential diagnoses lymphadenopathy and sarcoma, which are more common situations. The predominant histological type is pure seminoma (43%), followed by embryonic carcinoma (28%), teratocarcinoma (27%) and choriocarcinoma (2%)^3^. Surgical treatment is mandatory, with resection of intra-abdominal mass and chemotherapy may be an alternative, depending on the stage and histological type of malignant transformation^2^.
